# Developing Culturally Tailored Mobile Web App Education to Promote Breast Cancer Screening: Knowledge, Barriers, and Needs Among American Indian Women

**DOI:** 10.1007/s13187-022-02252-x

**Published:** 2023-01-11

**Authors:** Soonhee Roh, Yeon-Shim Lee

**Affiliations:** 1 Department of Social Work, University of South Dakota-Sioux Falls, 4801 North Career Ave, 145C, Sioux Falls, SD 57107 USA; 2 School of Social Work, San Francisco State University, 1600 Holloway Avenue, HSS 216, San Francisco, CA 94132 USA

**Keywords:** Breast cancer, Mammography, American Indian women, Mobile web app–based health intervention, mHealth

## Abstract

American Indian (AI) women face disproportionate rates of breast cancer mortality and cancer disparities. This study conducted qualitative research to assess perspectives of AI women towards breast cancer screening, knowledge, barriers, and needs about mobile web app–based education to promote breast cancer screening. This study, in collaboration with the Yankton Sioux Tribe (YST), followed a community-based participatory research approach and conducted two focus groups with a total of 22 YST women aged 40–70 years living on reservation in rural South Dakota. Each group consisted of 11 local professionals working in healthcare and social services and community members. A grounded theory was used for the qualitative analysis. A large portion of participants reported having prior knowledge about breast cancer and screening methods, yet lacked awareness of the detailed procedure and recommended guidelines. Competing priorities and cost of mammograms were noted as major barriers to screening. Participants wanted to learn—in a convenient and easy-to-understand manner—more about breast cancer and prevention from a credible source. Both groups were favorable toward novel educational tools, such as the mobile web app education, and cited potential health benefits, particularly for women aged 40s to 60s﻿. Our findings highlighted the importance of creating effective, culturally tailored educational interventions built into programs specific to AIs to increase understanding about breast cancer screening and promote screening behaviors among AI women. Particular attention to how AIs’ culture, beliefs, and barriers are implicated in screening behaviors could help with developing culturally tailored health education tools for this population.

## Introduction

Breast cancer is the most common form of cancer and the second leading cause of cancer death among women in the USA [1]. Roughly one in eight women will be diagnosed with breast cancer in their lifetime. In 2021, an estimated 281,550 women will be newly diagnosed with invasive breast cancer and about 43,600 women are expected to die from the disease [2]. Cancer is a large burden on the healthcare system—the economic expenses of breast cancer were projected to reach $20 billion by 2020 [3].

American Indian and Alaska Native (AI/AN) women shoulder disproportionate rates of rising incidence, cancer death, and low survival due to breast cancer, resulting in cancer health disparities. These disparities remain persistent with no sustained improvement. Breast cancer remains the second leading cause of cancer death for AI/AN women. Although AI/AN breast cancer incidence is lower than the incidence in non-Hispanic White (NHW) women, AI/AN women exhibit the highest breast cancer mortality and lowest breast cancer screening rates in the USA [4–8]. Sizable regional disparities in cancer incidences and mortality rates exist within the AI population [9]. AI women—particularly those residing in the Northern Plains—have the second highest cancer incident rate within the AI/AN population in the USA. Within the AI/AN population nationally, Northern Plains AI/ANs have the highest cancer mortality rate (275.5/100,000 population). This rate is higher than all races combined in the USA (200.9/100,000 population). When compared to their NHW regional counterparts and in the USA, Northern Plains AI/AN women (1) have an elevated risk of developing and/or dying from breast cancer and (2) are more likely to be diagnosed with advanced cancer. These rates indicate critical regional needs for interventions [10].

Early detection of breast cancer through regular mammograms can reduce breast cancer mortality up to 40% [11]. However, research examining the cancer screening patterns from 1997 to 2006 found no significant improvement in screening rates for Northern Plains AI women [9]. As a result, breast cancer remains the most common cause of cancer-related death in this population. Identified barriers to screening include low income and health insurance coverage, inadequate knowledge about breast cancer and screening guidelines, lack of need for screening in the absence of symptoms, geographic isolation, limited healthcare accessibility, and cultural stigma.

Mobile web app interventions are a new method with positive impact on constructive behavioral change; they have taken a primary place in numerous mobile health (mHealth) research initiatives (e.g., preventive cancer care, smoking cessation, weight management, alcohol consumption, and asthma monitoring and management) [12–15]. Mobile web apps, a powerful tool for educational and behavioral interventions, have been successfully applied in physical and mental health for adults of all ages [12, 16, 17, 18]. Moreover, the mobile web app–based intervention for breast cancer screening is a relatively low-cost way to reach large numbers of women. Available evidence suggests that socially, geographically isolated AIs may benefit the most from internet and mobile-based health/mental health interventions [19]. Lee et al. call for attention to culturally tailored services that address AIs’ unique barriers to using and adopting new digital technologies, such as friendlier computer interface and software design and improved usability and contents, as well as the physical environment where learning and computer usage take place [19]. Despite the significant breast cancer disparity, little intervention research has focused on this population. Moreover, to date, there is no information on whether a mobile web app–based intervention changes breast cancer screening behaviors among AI women.

The goal of this qualitative formative study was (1) to assess community member knowledge, barriers, and educational needs about breast cancer screening among the Northern Plains AI women; and (2) to explore how to best design education communication tools for rural, geographically isolated AI women to improve breast cancer screening behaviors, such as a mobile web app educational intervention. There have been some regional studies on AI women’s viewpoints about breast cancer screening, yet none has been conducted in the Northern Plains [20, 21]. Considering the heightened risk of the Northern Plains AI women and the importance of collecting region- and tribe-specific data, the Yankton Sioux Tribe (YST) in South Dakota (SD) served as the target population in this study.

## Methods

### Research Design

This study employed a community-based participatory research approach by building upon collaborative partnerships between the research team and the YST in SD. Our study entailed the creation of a community advisory board (CAB), which is a partnership with the local AI communities. The CAB consisted of eight individuals—AI healthcare professionals, social service providers, staff from the YST Bureau of Indian Services, and community and religious leaders—who guided us in all aspects of study development, implementation, and dissemination of research findings. The CAB convened quarterly to procure community support and involvement, provide important community insights about community concerns, assist in recruitment strategies, and ensure cultural relevance.

Two focus groups were undertaken in October 2020 to (1) elicit knowledge, cultural beliefs, and attitudes about breast cancer screening and (2) identify barriers and motivators for screening with YST women aged 40 to 70 years. The focus groups led to a broader discussion of culturally normative perspectives and a greater exchange of ideas regarding the main study themes than would be generated solely through individual interviews. Institutional Review Board approvals were obtained from the University of South Dakota and the YST.

### Sampling and Recruitment

This study used a purposive sampling technique to recruit a total of 22 participants in two focus groups (*N* = 22; *n* = 11 for each group). Each group represented one of the main stakeholder categories: YST community members and YST local professionals with backgrounds in cancer and health management (nurses, social service providers, and researchers). Dividing participants into two focus groups was intentional to capture the potential differences in their knowledge and awareness about and exposure to breast cancer. By incorporating into the research design a broader discussion of culturally normative perspectives in two similar, yet distinct, groups, this study sought to lay the groundwork for the development of culturally tailored interventions in AI communities. Eligibility criteria for participation in the focus groups included those (1) who were self-identified AI women of the YST, (2) who were aged 40 to 70 years, and (3) who resided on the YST reservation in SD. The participant age range of 40 to 70 was selected based on the breast cancer screening guidelines of the American Cancer Society that recommended women to begin regular mammograms at age 45 or at 40 if women opt to start screening earlier [22]. Participants were recruited through announcements on tribal public radios and local newspapers, flyers posted at community agencies and religious organizations, referrals, and by word of mouth. Research assistants screened interested individuals over the phone to determine whether they met the inclusion criteria. A total of 22 potential participants responded with interest, and all respondents were included. The final sample of 22 was determined by data saturation when no new information was generated from new participants.

### Data Collection

A semi-structured focus group guide was developed for this study based on a literature review, consultation with the CAB, and the authors’ past work with AIs [23]. The CAB reviewed the questions and probes, focusing on the wording and cultural appropriateness of each question, and ensured cultural relevance to AI women. The two focus group discussions were facilitated by the two authors with a bicultural and bilingual (English and Lakota) YST female research assistant. The research assistant was matched with respect to race/ethnicity and gender with participants and trained in discussion moderation and interviewing techniques. Each focus group session was conducted face-to-face in conference rooms at the YST reservation and lasted about 2 hours. Prior to the focus groups, all participants were fully informed about the purpose of the study, risk/benefits, confidentiality, and voluntary participation. Signed informed consent was completed by participants prior to the focus groups. Topics covered in the focus groups included (1) knowledge and beliefs about breast cancer and screening; (2) individual, structural, and cultural barriers to screening; (3) perspectives and needs about education communication tools, such as the mobile web app–based programs; and (4) suggestions on how to make the mobile web app–based interventions more culturally relevant and more acceptable to AI women. All sessions were audiotaped and transcribed verbatim by research assistants. Participants were provided $50 compensation and a gift card for costs of transportation.

### Data Analyses

Transcripts of two focus group sessions were reviewed for thematic and content analysis to identify salient themes. The study authors independently read and coded the transcripts for prominent themes that emerged iteratively in the text using Atlas 9 qualitative data analysis software. Following the procedures for grounded theory analysis [24, 25], the first step was to use inductive and open coding—a procedure for identifying, coding, and categorizing the emerging concepts occurring repeatedly throughout the data—to create data categories. Additionally, field notes and memoing techniques were used to record contextual details, emerging themes, and insights about substantive concepts and their theoretical relationships. Second, the authors identified and arranged commonly used words, phrases, and clusters of similar concepts into categories. Then, the authors developed a coding nomenclature based on the focus group transcripts. Frequencies of major coded responses were computed, and major themes were developed. Third, axial coding was conducted that identified the core concepts, regrouped the codes, and generated the relationships between the major analytical categories and subcodes. Coded text was analyzed to assess inter-coder reliability for each of the four areas addressed in the focus groups. Few to no differences were found. The most illustrative quotes representing the themes were selected from the transcripts.

## Results

As shown in Table 1, the age of participants ranged from 40 to 70, with a mean of 57.6 years. About 72.6% of participants have attended college. Most participants (77.3%) were born on a reservation and the majority (77.3%) of participants live with family members. Almost 41% of participants reported a monthly household income of less than $1499. Over half of participants (54.5%) were not employed. Participants were single and divorced in equal percentages (31.8%), while only 18.2% were married. Most respondents had a religious affiliation (95.5%); Protestant and Traditional Tribal Spirituality being the predominant ones (31.8% each). Most participants had medical insurance (86.4%). Many participants described their health as good or excellent (77.3%).

**Table 1:** Demographic characteristics among American Indian women (*N* = 22)

Age, M (SD)	Range: 40 to 70 years	57.6	(9.7)
Where born, *n* (%)	Reservation	17	(77.3)
	Non-reservation	5	(22.7)
Marital status, *n* (%)	Married	4	(18.2)
	Single (never married)	7	(31.8)
	Widowed	2	(9.1)
	Divorced	7	(31.8)
	Other (separated etc.)	2	(9.1)
Education, *n* (%)	High school or GED	6	(27.3)
	Some college or associate degree	14	(63.6)
	Bachelor’s degree	1	(4.5)
	Master’s degree	1	(4.5)
Living arrangement, *n* (%)	Alone	2	(9.1)
	With family members	17	(77.3)
	With both family and non-family members	2	(9.1)
	Other	1	(4.5)
Employed, *n* (%)	Yes	10	(45.5)
	No	12	(54.5)
Monthly household income﻿, *n* (%)	Under $500	2	(9.1)
	$501 to $999	3	(13.6)
	$1000 to $1499	4	(18.2)
	$1500 to $1999	4	(18.2)
	$2000 to $2499	3	(13.6)
	$3000 and over	6	(27.3)
Perceived health, *n* (%)	Poor	1	(4.5)
	Fair	4	(18.2)
	Good	15	(68.2)
	Excellent	2	(9.1)
Insurance, *n* (%)	Yes	19	(86.4)
	No	3	(13.6)
Religious affiliation, *n* (%)	Yes	21	(95.5)
	No	1	(4.5)

Focus group discussions with YST women yielded three key themes about breast cancer/screening for contextualized understanding of breast health behaviors among AI women: (1) knowledge about breast cancer/screening, (2) barriers to breast cancer screening, and (3) educational needs about breast cancer–related mobile web app–based education. These three themes emerged iteratively in the two focus groups and included a broad range of contextual sub-themes (Table ﻿2).

**Table 2:** Themes and sub-themes on breast cancer and screening among American Indian women (grounded)

Themes	Sub-themes	Codes and condensed meaning units
Knowledge about breast cancer and screening	Prior knowledge	• Primary source of information -Direct personal experience -Indirect experience (family, relatives, or someone participants knew) • Other sources (healthcare providers, media, brochures, and community events)
	Knowledge about symptoms	• Identified symptoms (pains, lumps, masses, changes in size or shape of the breasts)
	Knowledge about risk factors	• Individual factors -Demographics (e.g., age) -Health/mental health status (obesity, alcohol abuse, smoking) -Lifestyle (high fat diet, less physical exercise)
		• Familial factors -Family genetic history
		• Systemic factors -Life stressors (discrimination, oppression) -Environment (pesticide, pollution, or toxic chemicals that were prevalent on the reservation)
	Knowledge about breast cancer screening	• Breast self-exam (BSE) -Most frequently mentioned & practiced
		• Mammography -Mentioned in both groups, yet lacked knowledge on the specific guideline/procedure -Reluctancy related to radiation exposure
		• Clinical breast exam not surfaced in discussions
Barriers to breast cancer screening	Individual barriers	• Competing priorities (e.g., family and work) • Cost • Lack of transportation • Lack of adequate health insurance coverage • Prior negative experience with healthcare system • Fear of mammography or possible results
	Healthcare barriers	• Affordability • Available medical facilities for screening • Inadequate healthcare system • Mistrust of healthcare system • Sex of healthcare providers
	Institutional/systemic barriers	• Discrimination and oppression • Historical trauma • General lack of health/social resources
Educational needs related to breast cancer and screening (i.e., mobile web app education intervention)	Perceived benefits	• Increased knowledge and awareness • Convenience • Flexibility • Increased accessibility to breast health information • Real-time interaction and personalized messages • Reducing stigma • Global sense of helping the community • Benefitting future generations
	Perceived barriers	• Lack of internet access and mobile phones • Connectivity issues
	Suggestions for mobile web app–based interventions	• Contents -General educational messages related to breast cancer and screening (screening guideline and procedure, benefits, access to clinics/services) -Financial assistance and resources -Culturally tailored contents specific to American Indian communities (e.g., statistical data specific to American Indians, featuring indigenous music, clothes, and unique experience of American Indian women, Indigenous holistic wellness approach, intergenerational connectedness) -Preventive care and early education for young generation
		• Structure -Easy-to-understand, simple, interactive, and engaging format -Visual materials (videos, pictures, symbols) -Reminders -Variation in the duration of interventions -Variation in the frequency of receiving the educational messages (daily, weekly, monthly)

### Knowledge About Breast Cancer Screening

Focus group participants reported prior generic knowledge about breast cancer and screening. The majority thought that breast cancer is a serious health issue and believed that breast cancer causes death. They reported that their primary sources of breast cancer information came from healthcare providers or their personal experience with breast cancer and/or they have known someone who has had breast cancer (e.g., mother, sisters, relatives, and friends). Participants also obtained information on breast cancer and screening from various media sources, brochures, healthcare providers, and community events (e.g., campaigns).

Most participants identified breast cancer risk factors and symptoms (e.g., pains, lumps, mass, changes in size or shape of the breasts). Some participants attributed risk factors of breast cancer to modifiable behaviors (e.g., western style high fat diet, less physical exercise, obesity, alcohol abuse, and smoking), while others regarded risk factors to be an unavoidable consequence of family genetic history and age. Across all age groups, those who had a family member diagnosed with cancer were more willing to consider undergoing mammograms. Several participants expressed larger concerns that breast cancer might have been caused/exacerbated by a systemic factor, such as life stressors and environment. Participants varied in the emotions evoked by discussing breast cancer and screening, with most frequently expressing fear or worries about possible diagnosis of breast cancer. One woman said:

The family I grew up with nothing like that [breast cancer] was ever discussed, you know, no one ever talked about breast cancer or anything like that. When I started hearing about it all these scary things, you can die from it and the serious things that were going on. I am afraid to go get the mammogram because I thought what if I have this, and that’s how scared I was.

Overall, the YST women noted favorable attitudes towards breast cancer screening. Knowledge about breast cancer screening differed by type of screening. Participants most frequently mentioned breast self-examination (BSE) followed by mammography. A clinical breast examination did not surface in the focus group discussions. A higher proportion reported practicing regular BSE and knowing about its procedure and recommended frequency than for mammography. Women also expressed conflicting views about the role of mammograms, with some believing that mammography could be an effective diagnostic tool to detect breast cancer early, and others believing that BSE could be as effective as mammograms in diagnosis of cancer. Several participants talked about their own experiences with mammograms with some reporting extreme pains, and others being only moderately bothered. Some women were reluctant to regularly get a mammogram because they believed that the mammogram would cause radiation exposure, which may also cause cancer. The need for more adequate information about the specific procedures of mammograms, screening guidelines, or recommended age and frequency was prominent across both groups.

### Barriers to Breast Cancer Screening

Participants from both groups agreed that they usually do not seek help from a doctor unless they have a family history of cancer or have signs or symptoms of breast cancer. Multiple themes emerged across the two groups pertaining to barriers for breast cancer screening: competing demands, cost, lack of health insurance, lack of availability of screening facilities in the geographical area, absence of transportation, cultural stigma, fear of mammography or possible results, opposite sex practitioners, privacy concerns, mistrust of medical systems, and discrimination and oppression.

Most YST women indicated that due to preoccupation with multiple responsibilities of daily survival (work, childcare, caregiving issues), they were unable to make time to see a doctor or travel for screening. Cost was frequently cited as another key barrier to screening across both groups. Most focus group participants had limited income and indicated a mammogram is too expensive, especially for those who do not have adequate health insurance coverage. A lack of mammography service emerged repeatedly across the two groups. Access to screening was also affected by availability of clinics, transportation, and healthcare providers in the community. Many YST women living on the reservation, where screening facilities do not exist, must travel substantial distances to receive screening services. Some participants do not have reliable transportation to a clinic. Frustrations about the inadequacy of existing healthcare coverages emerged in all focus groups. A few participants related anecdotal accounts of misdiagnosis of breast cancer after having mammograms or lack of communication from their healthcare providers. Several participants reported being embarrassed or uncomfortable discussing or touching such a private body part during a mammogram, particularly with an opposite sex practitioner. However, most women agreed that the sex of practitioners was less important than “a good doctor” who they can trust. The prominent themes across the two groups were general lack of resources to address their health needs and associated vulnerability. Participants described instances of discrimination exacerbated by historical trauma (e.g., medical personnel trivializing their medical symptoms or dismissing their healthcare needs that required more careful medical exams).

### Educational Needs for the Mobile Web App Intervention

#### Perceived Benefits

Participants from both focus groups expressed strong agreement that the level of information about breast cancer and screening needs to be expanded. There was a variety of traditional and nontraditional approaches to promoting breast cancer screening discussed in the focus groups: grocery store bulletin boards, talking circles (a traditional method of sharing and discussing issues), brochure or pamphlets, educational DVD, media advertisements, campaigns, social media, and mobile reminders.

Across all groups, AI women referred to the use of innovative technology, such as a mobile web app–based education intervention for breast cancer screening and exposure to video content, as an effective medium for information dissemination about mammography. Participants in both groups were generally favorable toward the mobile web app intervention, and noted a potential health benefit, especially for women in their 40s to 60s﻿. Perceived benefits to participation in the mobile web app intervention included convenience (overcoming restrictions to place and time of delivery), flexibility by reducing traveling and waiting time, increased accessibility to important breast health information, real-time interaction, reducing stigma, personalized messages, and a global sense of helping community and future generations. As one woman in her mid-60s﻿ stated:

It would be really helpful because this is how our world is nowadays. I don’t have the time to go into hospital about myself, but with an app, you can do all that you can get information, probably interact with a doctor or something all over the app. Our phones are everything, we bank from them, send messages, do our work from these phones, so our phones are really our life phone.

One participant commented on the benefits of the mobile web app during the COVID era:

The education everything has to do with our health, now I’m finding many elderly add me on Facebook...I see a couple of them and they are lonesome we are not supposed to hug or anything [due to COVID], so if you offer this app to them, that will give them something to do for their mental health and help them focus on their health at that time.

A greater willingness to participate in the mobile web app intervention was associated with perceived direct benefits for participants or their communities, particularly benefitting future generations.

#### Perceived Barriers

Some women had concern about the lack of internet availability, mobile phones, and connectivity issues; however, others argued that the use of technology including internet, smartphones, and tablets has rapidly increased and Wi-Fi connections have improved on the YST reservation. In both focus group discussions, availability of the mobile web app educational service to community members focused on needing greater resources and funds. One participant noted:

Some people have a phone month to month and the next they don’t. What they did now is that the tribe can have free Wi-Fi, and what’s good about having free Wi-Fi is that many people who don’t have minutes, but they can use internet, Wi-Fi and apps. If they keep the Wi-Fi, that would be a good idea to promote the app. It takes grants and funds to secure the resource first.

#### Suggestions for the Mobile Web App Intervention

***Contents***: Across both focus groups, participants referred to the use of culturally tailored and YST community–specific health education content as critical for the development/implementation of the mobile web app intervention. Participants identified a range of information that need to be included in educational messages, such as breast cancer, breast cancer screening guidelines and procedures, screening benefits, and access to screening services and resources on or near the reservation (e.g., clinics and financial assistance for screening). Some participants desired statistics more relevant to them about incidence of breast cancer and survivorship in the YST community, which would possibly lead to greater participation in breast cancer screening. Participants spoke of the importance of contextualizing the “Indigenous holistic wellness approach” that is driven by sociocultural factors that influences health and health behaviors of AIs. Participants mentioned the integration of mind, body, and spirit, as well as one’s connection with oneself and others. For example, one woman in her 60s ﻿exemplified the Native way of the holistic wellness approach:

We come in contact with and within our environment. It’s the balance of our emotional, spiritual, and physical, how we live on a daily basis, is really important to us, women we take care of our whole families, in order to take care of them, we need to take care of ourselves, our bodies, our mental awareness, so we can live our healthy lives, so a lot of those factors contribute to our wellness and our health.

The importance of prevention and early education for younger generations was a theme across the two focus groups. Multiple participants expressed concern about a lack of education specific to younger generations. Some attributed it to the fact that they were not of the recommended screening age, or that healthcare providers might not have supplied information about breast cancer. Participants suggested that as younger people, perhaps not ready for screening, are more familiar with a novel technology than participants aged 40 to 70, they would be more likely to find the mobile web app intervention a useful tool that may increase awareness of breast cancer screening at an early age and undergo their mammography in the right time frame. Participants also suggested creating educational messages involving young girls and intergenerational connectedness to encourage future screening in the web app program.

**Structure**: Most participants felt that the mobile web app education intervention for breast cancer screening should be developed in an easy-to-understand, simple, interactive, and engaging manner. Some participants indicated that reminders and visual materials (e.g., videos, pictures, symbols) would be effective in the mobile web app intervention in prompting participation in breast cancer screening. Participants expressed different opinions regarding the duration of the mobile web app intervention; some desired a shorter period of 7 days, while others preferred a 2- to 4-week program. There was a wide variation regarding the frequency of receiving the educational messages: per day, per week, and per month. Most participants preferred to receive the messages during evening hours based on their work schedules or other commitments.

## Discussion and Implications

The present study explored knowledge, barriers, and educational needs about breast cancer screening with a focus on the novel mobile web app intervention among 22 YST women aged 40 to 70 years residing on reservation in rural areas in SD.

Our key finding indicated that novel education programs, such as the mobile web app intervention for breast cancer screening, which are culturally tailored and built into programs specific to AIs, can be a feasible, acceptable, and effective tool to improve participation in breast cancer screening among AI women. All participants desired additional education about breast cancer. Most participants indicated the high usage of internet technology and mobile phone–based apps to access health/mental health–related and non-health-related information and to maintain social connections. Participants were generally favorable toward the mobile web app education intervention, and noted a potential health benefit, particularly for women in their 40s to 60s. Participants wanted to learn more about breast cancer, screening methods, and prevention from a credible source (e.g., healthcare providers, educational materials, and media sources) and in a convenient, easy-to-understand, and engaging manner. Results showed that interactive multimedia messages and visual materials (e.g., videos, pictures, visual images) are more likely to capture AI women’s attention and to motivate them to engage.

The YST women felt that the mobile web app education intervention should contain information relevant to Indigenous populations. They desired culturally tailored information, which would lead to more breast cancer screening. Cultural relevance entails (1) putting pictures and music related to the YST on the app, (2) providing statistics and information about local clinics for screening tests specific to the YST community, (3) incorporating the Indigenous holistic wellness approach, and (4) bolstering intergenerational connectedness and early education for younger generations. While most participants were comfortable with using the mobile devices, several noted issues concerning the availability and connectivity of Wi-Fi as a potential barrier that might hinder some women from participating in the mobile web app education intervention. Thus, intervention efforts should address concrete improvements in those areas that would significantly influence the ability to access educational resources. Comprehensive funding that builds infrastructure and encourages internet service providers to invest in rural and remote areas of reservations is critical to address healthcare and social needs.

Most participants had prior generic knowledge about breast cancer screening, and agreed that mammography is crucial to the health of their community. Participants of this study were aged 40 to 70 and most had their own experiences with mammography or knew someone who had breast cancer. Despite the struggle for daily survival due to a lack of economic and social resources, participants placed a great value on the breast health of their community, especially if applied to the improvement of health of the younger generation. They expressed a strong interest in learning more about preventing the onset of breast cancer and breast cancer screening. Findings indicated that people would be more willing to receive breast cancer screening if they have had a personal experience of a family member affected by breast or other types of cancer; and they have a more favorable attitude toward the potential benefits of screening.

One of the focus group discussions centered on barriers to accessing breast cancer screening. The most important barriers included competing priorities, high cost of mammography, lack of health insurance coverage, lack of availability of screening facilities in the geographical area, transportation, and culturally inappropriate screening services. Similar barriers have been found in prior studies [21]. This study indicated that socioeconomic needs take priority over health among AI women. Basic survival needs of food, housing, and expendable income were of paramount importance. Increased access to healthcare and more health information about health management and prevention were viewed as important, but secondary to basic needs of living. Our findings are consistent with previous studies that indicated similar barriers among AIs, African Americans, Latinos, and other people living in poverty-stricken areas [12, 21]. More important barriers might be related to deep-seated mistrust of medical systems and long-standing oppression and discrimination, highlighting the core importance of building trust over time. Results showed that AI women would be more motivated to participate in breast cancer screening if their concerns were adequately addressed and if barriers to participation were ameliorated.

## Limitations and Conclusions

This study has several limitations. The small number of participants with the use of convenience sampling was the major limitation to our study. Participants were women from the YST aged 40 to 70 who live in a small geographic location of the Northern Plains. The findings, therefore, cannot be generalized to AI groups in different tribes, regions, and ages. The complexities of health beliefs and behaviors should be taken into account, as well as the unique features of the YST group.

Despite these limitations, this study contributed to the limited body of literature on AI women’s knowledge, beliefs, attitudes, barriers, and educational needs about breast cancer and screening for the disease. Identifying these themes provided guidance for designing the innovative mobile web app–based educational intervention multimedia messaging program, with particular attention to breast health literacy and cultural humility. To our knowledge, this is the first study to elucidate the Northern Plains AI women’s perspectives and experiences about breast cancer and screening to assess the educational needs and concerns. Given the widespread use of mobile technology, the mobile web app intervention provides a viable mode of service delivery that builds upon low-cost technology and enhances accessibility and sustainability of health intervention to help reduce cancer health disparities experienced in Indian country. While our study is region- and tribe-specific, it may have implications for other regional and tribal initiatives for breast cancer screening and health educational communication. Future investigation that tests the efficacy and cultural acceptability of these tools with various AI subgroups is warranted.

## References

[1] DeSantis CE, Ma J, Gaudet MM, Newman LA, Miller KD, Sauer AG, Jemal A, Siegel RL (2019). Breast cancer statistics. CA Cancer J Clin.

[2] American Cancer Society (2021) Breast cancer facts and figures in brief. https://www.cancer.org/research/acs-research-highlights/breast-cancer-research-highlights.html. Accessed 31 Dec 2021

[3] Greenup RA, Rushing C, Fish L, Campbell BM, Tolnitch L, Hyslop et al (2021) Financial costs and burden related to decision for breast cancer surgery. J Oncol Pract 15(8):e666-e676 10.1200/JOP.18.00796 PMC7846052 31356147

[4] Burnett-Hartman AN, Adams SV, Bansal A, McDougall JA, Cohen SA, Karnopp A, Warren-Mears V, Ramsey SD (2018). Indian Health Service care system and cancer stage in American Indians and Alaska Natives. J Health Care Poor Underserved.

[5] Roen EL, Roubidoux MA, Joe AI, Russell TR, Soliman AS (2013). Adherence to screening mammography among American Indian women of the Northern Plains. Breast Cancer Res Treat.

[6] Espey DK, Jim MA, Cobb N, Bartholomew M, Becker T, Haverkamp D, Plescia M (2014). Leading causes of death and all-cause mortality in American Indians and Alaska Natives. Am J Public Health Res.

[7] Roubidoux MA, Kaur JS, Rhoades DA (2022). Health disparities in cancer among American Indians and Alaska Natives. Acad Radiol.

[8] White A, Richardson LC, Ekwueme DU, Kaur JS (2014). Breast cancer mortality among American Indian and Alaska Native women, 1990–2009. Am J Public Health.

[9] Watanabe-Galloway S, Flom N, Xu L, Duran T, Frerichs L, Kennedy F, Smith CB, Jaiyeola AO (2011). Cancer-related disparities and opportunities for intervention in Northern Plains American Indian communities. Public Health Rep.

[10] Roh S, Burnette CE, Lee YS, Jun JS, Lee YH, Lee KH (2018). Breast cancer literacy and health beliefs related to breast cancer screening among American Indian women. Soc Work Health Care.

[11] Mammography facts (2017) American College of Radiology Accreditation. https://www.mammographysaveslives.org/Facts.aspx. Accessed 31 Dec 2021

[12] Lee H, Ghebre R, Le C, Jang YJ, Sharratt M, Yee D (2017) Mobile phone multilevel and multimedia messaging intervention for breast cancer screening: pilot randomized controlled trial. JMIR Mhealth and Uhealth 5(11):e154 10.2196/mhealth.7091 PMC5698632 29113961

[13] Hartman SJ, Nelson SH, Cadmus-Bertram LA, Patterson RE, Parker BA, Pierce JP (2016). Technology- and phone-based weight loss intervention: pilot RCT in women at elevated breast cancer risk. Am J Prev Med.

[14] Cole-Lewis H, Kershaw T (2010). Text messaging as a tool for behavior change in disease prevention and management. Epidemiol Rev.

[15] Fjeldsoe BS, Marshall AL, Miller YD (2009). Behavior change interventions delivered by mobile telephone short-message service. Am J Prev Med.

[16] Krishna S, Boren SA, Balas EA (2009). Healthcare via cell phones: a systematic review. Telemed J E Health.

[17] Kumar S, Nilsen WJ, Abernethy A, Atienza A, Patrick K, Pavel M (2013). Mobile health technology evaluation: the mHealth evidence workshop. Am J Prev Med.

[18] Riley WT, Rivera DE, Atienza AA, Nilsen W, Allison SM, Mermelstein R (2011). Health behavior models in the age of mobile interventions: are our theories up to the task?. Transl Behav Med.

[19] Lee YS, Roh S, Donahue SR, Lee KH, Kim S (2017). Technology acceptance among American Indian older adults: the role of perceived health, social engagement, and social support. J Sociol Social Work.

[20] Daley CM, Kraemer-Diaz A, James AS, Monteau D, Joseph S, Pacheco J, Bull JW, Cully A, Choi WS, Greiner KA (2012). Breast cancer screening beliefs and behaviors among American Indian women in Kansas and Missouri: a qualitative inquiry. J Cancer Educ.

[21] Filippi MK, Ndikum-Moffor F, Braiuca SL, Goodman T, Hammer TL, James AS, Choi WS, Greiner KA, Daley CM (2013). Breast cancer screening perceptions among American Indian women under age 40. J Cancer Educ.

[22] American Cancer Society (2021) Breast cancer screening guidelines. https://www.cancer.org/latest-news/special-coverage/american-cancer-society-breast-cancer-screening-guidelines.html. Accessed 31 Dec 2021

[23] Roh S, Burnette CE, Lee YS (2018). Prayer and faith: spiritual coping among American Indian women cancer survivors. Health Soc Work.

[24] Strauss MA (1987). Qualitative analysis for social scientists.

[25] Strauss AL, Corbin J (1990). Basics of qualitative research: grounded theory procedures and techniques.

